# Guideline-recommended inflammatory testing in symptomatic dermographism: a real-world study

**DOI:** 10.3389/fimmu.2026.1901562

**Published:** 2026-08-06

**Authors:** Mojca Bizjak-Suran, Larisa Stojanovič

**Affiliations:** 1 Division of Allergy, University Clinic of Respiratory and Allergic Diseases Golnik, Golnik, Slovenia; 2 Faculty of Medicine, University of Ljubljana, Ljubljana, Slovenia; 3 Faculty of Medicine, University of Maribor, Maribor, Slovenia

**Keywords:** chronic inducible urticaria, chronic spontaneous urticaria, complete blood count, C-reactive protein, inflammatory biomarkers, real-world cohort, serum protein electrophoresis, symptomatic dermographism

## Abstract

**Introduction:**

Symptomatic dermographism (SD) is the most common form of chronic inducible urticaria and is increasingly recognized as a burdensome disease. The 2026 international urticaria guideline recommends eliciting dermographism and threshold testing for diagnosis and includes differential blood count and erythrocyte sedimentation rate or C-reactive protein (CRP) as an extended diagnostic work-up in patients with SD. However, the clinical usefulness of these inflammatory tests in SD remains incompletely characterized.

**Methods:**

In this retrospective real-world cohort study, we examined whether routine inflammatory markers provide interpretable signals in SD, using chronic spontaneous urticaria (CSU) as a reference comparator and serum protein electrophoresis-derived markers, including the inflammatory protein ratio (IPR) ([alpha-1 + alpha-2]/albumin × 100), as exploratory measures of inflammation. We included 726 patients with available serum protein electrophoresis (SPE) data: 579 with CSU only, 107 with SD only, and 40 with both diagnoses. Patients with both CSU and SD were excluded from the primary binary comparison, leaving 686 patients for the main CSU-only versus SD-only analysis.

**Results:**

Compared with CSU, SD showed lower CRP (median [IQR]: 1.40 [0.70–3.30] vs. 2.10 [0.90–5.50] mg/L; *p* = 0.003) and higher basophil counts (0.04 [0.03–0.05] vs. 0.03 [0.02–0.05] × 10^9^/L; *p* < 0.001), both of which remained significant after false discovery rate correction. SD patients were younger than CSU patients (39.0 [32.0–49.0] vs. 45.0 [35.0–58.0] years; *p* = 0.001). In age- and sex-adjusted logistic regression models, higher CRP and lower basophils remained associated with CSU. IPR did not differ significantly between SD and CSU but correlated strongly with CRP in both SD- and CSU-only patients (rho = 0.551 and rho = 0.550, respectively; both *p* < 0.001).

**Discussion:**

Overall, these results show that some interpretable inflammatory signals are present in SD, but they do not clearly explain why these markers should be measured routinely in SD.

## 1 Introduction

Symptomatic dermographism (SD), also referred to as urticaria factitia or dermographic urticaria, is a form of chronic inducible urticaria (CIndU) triggered by mechanical shear stress, such as rubbing, scratching, friction, or stroking, applied parallel to the skin surface (1). Recent international PREVALENCE-D data estimate an adjusted point prevalence of SD of 3.20% and a lifetime prevalence of 5.94%, supporting the view that SD is common (2). Earlier clinical data also showed that SD is often persistent, with a mean disease duration of approximately 6 years, and that it can cause substantial quality-of-life impairment (3).

The pathogenesis of SD remains incompletely understood. Current evidence supports mast cells as central effector cells, but the upstream mechanisms that activate them after mechanical shear stress remain unclear. Emerging data suggest that SD may involve heterogeneous pathways, including immunoglobulin E (IgE)-dependent and IgE-independent mast-cell activation, neurogenic mechanisms, microbiome-related changes, and candidate serum microRNAs (1). These findings support the view that SD is more than a purely mechanical skin reaction. At the same time, these mechanistic insights do not define whether routine blood-based inflammatory markers are clinically useful in SD.

The 2026 international urticaria guideline recommends provocation testing to elicit dermographism and assess provocation thresholds. It also includes differential blood count and erythrocyte sedimentation rate (ESR) or C-reactive protein (CRP) as an extended diagnostic work-up guided by clinical history (4). However, it remains unclear what these inflammatory tests add to the assessment of SD or whether they provide interpretable information beyond helping to exclude alternative diagnoses or detect systemic inflammation.

Serum protein electrophoresis (SPE) is not routinely recommended in the diagnostic evaluation of chronic urticaria. However, it may be useful in selected patients with atypical urticarial presentations or systemic inflammatory features, including fever, leukocytosis, persistently high CRP, bone pain, or systemic symptoms, particularly when monoclonal gammopathy-associated conditions such as Schnitzler syndrome are considered in the differential diagnosis (5, 6). SPE separates serum proteins into albumin and different globulin fractions, including alpha, beta, and gamma fractions. Monoclonal spike-like peaks suggest monoclonal gammopathy, whereas polyclonal changes may reflect reactive or inflammatory processes (7). Albumin behaves as a negative acute-phase reactant, whereas alpha-1 and alpha-2 fractions contain several acute-phase proteins and may increase during inflammatory responses (7). In 2022, the inflammatory protein ratio (IPR), calculated as ([alpha-1 + alpha-2]/albumin) × 100, was proposed as a potential SPE-derived marker of inflammatory status (8).

In this study, we evaluated the clinical usefulness of guideline-recommended inflammatory testing in SD, focusing on CRP and differential blood count. Chronic spontaneous urticaria (CSU) was used as a clinically related and better-characterized reference comparator, and SPE-derived markers were analyzed as exploratory measures of inflammation. The aim was to determine whether low-cost, routine markers provide interpretable inflammatory signals in SD.

## 2 Materials and methods

### 2.1 Study design and participants

We conducted a retrospective, real-world cohort study of patients diagnosed with SD or CSU at the University Clinic of Respiratory and Allergic Diseases Golnik between January 2016 and April 2026. Eligible patients had available SPE data and other variably available routine inflammatory markers. The study was approved by the Slovenian National Medical Ethics Committee (KME78/09/14), and all participants provided written informed consent.

### 2.2 Clinical evaluation

CSU and SD were diagnosed according to clinical history and guideline-based criteria (4, 9). SD was confirmed using the FricTest 4.0, a standardized provocation tool (10), which was also used in CSU patients to exclude concomitant SD. Conditions mimicking chronic urticaria, including suspected urticarial vasculitis, autoinflammatory syndromes, and bradykinin-mediated angioedema, were excluded based on clinical evaluation (4).

### 2.3 Laboratory parameters

Blood samples were taken during routine care at the first visit, and laboratory tests were conducted at the Laboratory for Clinical Biochemistry and Hematology, University Clinic Golnik. Available tests included automated complete blood count (CBC) with differential, SPE, CRP, total serum protein, serum immunoglobulins IgA, IgG, and IgM, and anti-thyroid peroxidase antibodies (anti-TPO). Absolute values of CBC with differential and SPE parameters were collected (**Table 1**). Leukocytosis was defined as a leukocyte count > 10.00 × 10^9^/L and neutrophilia as an absolute neutrophil count > 7.40 × 10^9^/L, based on institutional laboratory reference ranges. High CRP was defined as ≥ 5.0 mg/L, and anti-TPO positivity as ≥ 34 IU/mL (11).

**Table 1:** Demographic and key laboratory parameters.

Parameter	Overall (*n* = 686)	SD-only (*n* = 107)	CSU-only (*n* = 579)	*p-*value	FDR-adjusted *p*-value
Demographics					
Age (years)	45.0 (35.0–57.0)	39.0 (32.0–49.0)	45.0 (35.0–58.0)	**0.001**	**0.009**
Female sex	453 (66.0%)	77 (72.0%)	376 (64.9%)	0.183	0.251
CBC and CRP					
Leukocytes (× 10^9^/L)	6.64 (5.57–8.12), *n* = 445	6.66 (5.61–8.02), *n* = 79	6.64 (5.56–8.21), *n* = 366	0.701	0.742
Neutrophils (× 10^9^/L)	4.00 (3.26–5.24), *n* = 445	3.93 (3.03–5.05), *n* = 79	4.04 (3.30–5.24), *n* = 366	0.197	0.251
Lymphocytes (× 10^9^/L)	1.79 (1.44–2.22), *n* = 445	1.90 (1.52–2.24), *n* = 79	1.76 (1.42–2.22), *n* = 366	0.209	0.251
Eosinophils (× 10^9^/L)	0.12 (0.07–0.20), *n* = 445	0.14 (0.09–0.22), *n* = 79	0.12 (0.07–0.20), *n* = 366	0.188	0.251
Basophils (× 10^9^/L)	0.03 (0.02–0.05), *n* = 445	0.04 (0.03–0.05), *n* = 79	0.03 (0.02–0.05), *n* = 366	**< 0.001**	**< 0.009**
CRP (mg/L)	2.00 (0.80–5.10), *n* = 603	1.40 (0.70–3.30), *n* = 99	2.10 (0.90–5.50), *n* = 504	**0.003**	**0.018**
High CRP	153 (25.4%), *n* = 603	16 (16.2%), *n* = 99	137 (27.2%), *n* = 504	**0.023**	0.083
SPE-related markers					
Albumin (g/L)	43.60 (41.68–45.40), *n* = 654	43.55 (41.63–45.40), *n* = 100	43.60 (41.68–45.43), *n* = 554	0.852	0.852
Alpha-1 (g/L)	2.90 (2.60–3.20), *n* = 654	2.80 (2.60–3.10), *n* = 100	2.90 (2.60–3.20), *n* = 554	0.058	0.142
Alpha-2 (g/L)	6.90 (6.30–7.60), *n* = 654	6.80 (6.10–7.50), *n* = 100	7.00 (6.30–7.70), *n* = 554	0.117	0.211
Derived markers					
NLR	2.33 (1.77–3.04), *n* = 445	2.15 (1.55–2.79), *n* = 79	2.35 (1.78–3.12), *n* = 366	**0.043**	0.129
High NLR	179 (40.2%), *n* = 445	28 (35.4%), *n* = 79	151 (41.3%), *n* = 366	0.377	0.424
SII	601.4 (435.3–807.1), *n* = 445	533.12 (410.06–818.75), *n* = 79	614.72 (441.84–801.75), *n* = 366	0.194	0.251
CRP-to-albumin ratio	0.05 (0.02–0.12), *n* = 576	0.03 (0.02–0.08), *n* = 93	0.05 (0.02–0.13), *n* = 483	**0.007**	**0.032**
IPR (%)	22.38 (20.28–25.42), *n* = 654	21.59 (19.37–25.27), *n* = 100	22.47 (20.51–25.45), *n* = 554	0.097	0.194
Thyroid autoimmunity					
Anti-TPO positivity	74 (16.3%), *n* = 455	7 (9.0%), *n* = 78	67 (17.8%), *n* = 377	0.063	0.142

Data are presented as median (IQR) or *n* (%). Where data were not available for all patients, the number of patients with available data is shown. Definitions of derived inflammatory markers are provided in the Methods section. *p*-values were calculated using Fisher’s exact test for categorical variables and the Mann–Whitney *U* test for continuous variables. FDR-adjusted *p*-values were calculated using the Benjamini–Hochberg method across all comparisons shown. High CRP was defined as CRP ≥ 5 mg/L, high NLR as NLR ≥ 2.5, and anti-TPO positivity as anti-TPO ≥ 34 IU/mL. *anti-TPO*, antithyroid peroxidase antibodies; *CBC*, complete blood count; *CRP*, C-reactive protein; *CSU*, chronic spontaneous urticaria; *FDR*, false discovery rate; *IPR*, inflammatory protein ratio; *IQR*, interquartile range; *NLR*, neutrophil-to-lymphocyte ratio; *SD*, symptomatic dermographism; *SII*, systemic immune-inflammation index; *SPE*, serum protein electrophoresis.

Bold values indicate statistically significant results (*p* < 0.05).

CBC with differential was performed using automated Sysmex hematology analyzers (XN 3100). SPE was performed by capillary electrophoresis using Sebia systems (MiniCap Flex Piercing). CRP, total serum protein, serum immunoglobulins, and anti-TPO were measured using automated Roche laboratory platforms (Cobas Pro, Cobas Pure).

The following inflammatory ratios were calculated: neutrophil-to-lymphocyte ratio (NLR; neutrophil count/lymphocyte count), platelet-to-lymphocyte ratio (PLR; platelet count/lymphocyte count), monocyte-to-lymphocyte ratio (MLR; monocyte count/lymphocyte count), systemic immune-inflammation index (SII; neutrophil count × platelet count/lymphocyte count), systemic inflammation response index (SIRI; neutrophil count × monocyte count/lymphocyte count), CRP-to-albumin ratio (CRP/albumin), and IPR ([(alpha-1 + alpha-2)/albumin] × 100) (8, 12).

### 2.4 Statistical analysis

Analyses were performed using IBM SPSS Statistics version 25. Continuous variables are reported as medians and interquartile ranges (IQR), and categorical variables as numbers and percentages. For the primary SD-only versus CSU-only comparison, continuous variables were compared using the Mann–Whitney *U* test and categorical variables using Fisher’s exact test. For the primary SD-only versus CSU-only comparison, false discovery rate (FDR)-adjusted *p*-values were calculated using the Benjamini–Hochberg method across all unadjusted group comparisons shown in **Table 1**. Associations between laboratory markers were examined separately in SD- and CSU-only patients using Spearman correlation with pairwise available data. Correlation strength was interpreted as weak (rho = 0.10–0.29), moderate (rho = 0.30–0.50), and strong (rho > 0.50) (11). Selected markers were then analyzed in separate exploratory logistic regression models adjusted for age and sex. These models described age- and sex-adjusted associations with the diagnostic group and were not intended as multivariable prediction models; no formal predictive performance assessment was performed. CSU-only status was coded as the outcome. Skewed nonnegative continuous markers were log1p-transformed and standardized before modeling. Binary markers were modeled as present versus absent. Two-sided *p*-values below 0.05 were considered statistically significant.

## 3 Results

### 3.1 SD-only patients were younger than CSU-only patients

Among 726 patients, 40 had both SD and CSU and were excluded from the primary binary comparison. The main analysis therefore included 686 patients: 107 with SD only and 579 with CSU only. SD-only patients were younger than CSU-only patients [median (IQR): 39.0 (32.0–49.0) vs. 45.0 (35.0–58.0) years; *p* = 0.001], whereas the sex distribution did not differ significantly between groups [female sex: 77/107 (72.0%) vs. 376/579 (64.9%); *p* = 0.183] (**Table 1**).

### 3.2 SD showed lower CRP and higher basophil counts than CSU

Compared with CSU-only patients, SD-only patients had lower CRP [1.40 (0.70–3.30) mg/L vs. 2.10 (0.90–5.50) mg/L; *p* = 0.003; FDR-adjusted *p* = 0.018] and higher basophil counts [0.04 (0.03–0.05) × 10^9^/L vs. 0.03 (0.02–0.05) × 10^9^/L; *p* < 0.001; FDR-adjusted *p* < 0.009]. Leukocyte, neutrophil, lymphocyte, and eosinophil counts did not differ significantly between groups. The prevalence of high CRP was lower in SD-only than in CSU-only patients [16/99 (16.2%) vs. 137/504 (27.2%); *p* = 0.023; FDR-adjusted *p* = 0.083], and NLR was lower in SD-only than in CSU-only patients [2.15 (1.55–2.79) vs. 2.35 (1.78–3.12); *p* = 0.043; FDR-adjusted *p* = 0.129]; however, these differences were significant only in the unadjusted comparisons and not after FDR correction (**Table 1**; **Supplementary Table S1**).

### 3.3 Most SPE-related and derived markers did not differ significantly between SD and CSU

Albumin, alpha-1, and alpha-2 concentrations did not differ significantly between SD- and CSU-only patients. IPR was slightly lower in SD-only than in CSU-only patients, but the difference did not reach statistical significance [21.59% (19.37%–25.27%) vs. 22.47% (20.51%–25.45%); *p* = 0.097]. The CRP-to-albumin ratio was lower in SD-only than in CSU-only patients [0.03 (0.02–0.08) vs. 0.05 (0.02–0.13); *p* = 0.007; FDR-adjusted *p* = 0.032]. Anti-TPO positivity was numerically less frequent in SD-only than in CSU-only patients [7/78 (9.0%) vs. 67/377 (17.8%); *p* = 0.063], but this difference did not reach statistical significance.

### 3.4 Exploratory adjusted models supported higher CRP and lower basophils as CSU-associated signals

As SD-only patients were younger than CSU-only patients, exploratory age- and sex-adjusted logistic regression models were fitted for selected markers (**Table 2**). Higher CRP remained associated with CSU (adjusted OR: 1.50 per 1 standard deviation increase after log1p transformation; 95% CI = 1.15–1.96; *p* = 0.003). The CRP-to-albumin ratio was also associated with CSU (adjusted OR: 2.10; 95% CI = 1.24–3.57; *p* = 0.006). Higher basophil counts were associated with lower odds of CSU, consistent with higher basophils in SD (adjusted OR: 0.62; 95% CI = 0.49–0.80; *p* < 0.001). Other selected markers, including leukocytes, neutrophils, NLR, SII, and IPR, were not significantly associated with the diagnostic group after age and sex adjustment.

**Table 2:** Age- and sex-adjusted logistic regression models comparing CSU with SD.

Marker	*n*	Adjusted OR for CSU (95% CI)	*p*-value
Leukocytes	445	1.13 (0.88–1.45)	0.343
Neutrophils	445	1.24 (0.96–1.59)	0.095
Basophils	445	0.62 (0.49–0.80)	**< 0.001**
CRP	603	1.50 (1.15–1.96)	**0.003**
High CRP	603	1.94 (1.09–3.45)	**0.024**
NLR	445	1.28 (0.97–1.68)	0.085
High NLR	445	1.16 (0.69–1.94)	0.577
SII	445	1.23 (0.95–1.59)	0.121
CRP-to-albumin ratio	576	2.10 (1.24–3.57)	**0.006**
IPR	654	1.23 (0.96–1.56)	0.099
Anti-TPO positivity	455	2.43 (1.06–5.59)	**0.036**

Each marker was analyzed in a separate exploratory logistic regression model adjusted for age and sex. The models were used to describe age- and sex-adjusted associations with diagnostic group and were not intended as multivariable prediction models; no formal predictive performance assessment was performed. The dependent variable was diagnostic group, with CSU-only patients coded as the outcome relative to SD-only patients. Odds ratios above 1 indicate higher odds of CSU, whereas odds ratios below 1 indicate higher odds of SD. Continuous markers were log1p-transformed and standardized before modeling. Binary markers were modeled as present versus absent. High CRP was defined as CRP ≥ 5 mg/L, high NLR as NLR ≥ 2.5, and anti-TPO positivity as anti-TPO ≥ 34 IU/mL. *anti-TPO*, antithyroid peroxidase antibodies; *CI*, confidence interval; *CRP*, C-reactive protein; *CSU*, chronic spontaneous urticaria; *IPR*, inflammatory protein ratio; *NLR*, neutrophil-to-lymphocyte ratio; *OR*, odds ratio; *SD*, symptomatic dermographism; *SII*, systemic immune-inflammation index.

Bold values indicate statistically significant results (*p* < 0.05).

### 3.5 IPR correlated strongly with CRP in both diagnostic groups

Correlations with CRP were examined separately within SD- and CSU-only patients (**Table 3**). CRP correlated strongly with IPR in both SD-only patients (rho = 0.551; *p* < 0.001) and CSU-only patients (rho = 0.550; *p* < 0.001). Albumin showed the expected inverse correlation with CRP in both groups (rho = − 0.357 in SD-only and rho = − 0.316 in CSU-only; both *p* < 0.001). CRP-cellular-marker coupling appeared more consistent in CSU-only patients, where CRP correlated with NLR, SII, neutrophils, and inversely with basophils. In SD-only patients, CRP correlated with NLR and SII, while correlations with neutrophils and basophils were not statistically significant.

**Table 3:** Spearman correlations of CRP with selected inflammatory markers within CSU- and SD-only patients.

Marker correlated with CRP	SD-only	CSU-only
IPR (%)	0.551; *n* = 93; *p* < 0.001	0.550; *n* = 483; *p* < 0.001
Albumin (g/L)	− 0.357; *n* = 93; *p* < 0.001	− 0.316; *n* = 483; *p* < 0.001
NLR	0.232; *n* = 74; *p* = 0.047	0.252; *n* = 335; *p* < 0.001
SII	0.273; *n* = 74; *p* = 0.019	0.317; *n* = 335; *p* < 0.001
Neutrophils (× 10^9^/L)	0.207; *n* = 74; *p* = 0.076	0.371; *n* = 335; *p* < 0.001
Basophils (× 10^9^/L)	− 0.083; *n* = 74; *p* = 0.482	− 0.297; *n* = 335; *p* < 0.001

Values are Spearman rho, available *n*, and *p*-value. Correlations were calculated separately within SD- and CSU-only patients using pairwise available data. *CRP*, C-reactive protein; *CSU*, chronic spontaneous urticaria; *IPR*, inflammatory protein ratio; *NLR*, neutrophil-to-lymphocyte ratio; *SD*, symptomatic dermographism; *SII*, systemic immune-inflammation index.

## 4 Discussion

In this real-world cohort, guideline-recommended inflammatory testing in SD showed limited but interpretable group-level signals. Compared with CSU, SD was associated with lower CRP and higher basophil counts, both of which remained significant after FDR correction. Most other complete blood count variables, derived hematologic ratios, and SPE-derived markers did not clearly distinguish SD from CSU. These findings suggest that routine inflammatory markers may help characterize modest differences in systemic inflammation between the groups.

The lower CRP signal in SD is consistent with earlier comparative work. Can and Kocatürk reported that SD patients were younger, had lower ESR and CRP, and less frequently reported systemic symptoms such as arthralgia or malaise than CSU patients (13). In the present cohort, the CRP difference appeared both as a lower median CRP and as a lower prevalence of high CRP. After adjustment for age and sex, higher CRP remained associated with CSU. These findings support the interpretation that CSU, used here as a clinically related and better-characterized comparator, exhibits a stronger systemic inflammatory signal than SD. Importantly, SPE-derived parameters were not reported in previous comparative SD-CSU studies, which provided the rationale for examining SPE-based inflammatory measures in the present analysis.

The basophil finding remained significant after age and sex adjustment. Basophil counts were higher in SD than in CSU and were associated with lower odds of CSU. This may reflect the broader observation that CSU can be accompanied by basopenia, particularly in patients with an autoimmune phenotype (11, 14). However, the absolute difference in basophil counts was small, and routine automated basophil counts have methodological limitations at very low values (15). Basophil counts should therefore be interpreted as a group-level signal rather than a clinically useful discriminator for individual patients.

Simple CBC-derived inflammatory ratios added little beyond CRP and basophil count in the SD-CSU comparison. NLR was lower in SD than in CSU in the unadjusted comparison, but this association was not significant after age and sex adjustment. SII, leukocyte counts, and neutrophil counts also did not clearly differ between the groups. This is consistent with Metin et al., who found no significant differences in NLR, PLR, CRP, or ESR between SD patients and healthy controls (16). By contrast, Weissmann et al. reported higher NLR and PLR in CSU and among patients with severe CSU, suggesting that hematologic ratios may be more informative in CSU than in SD (17).

SPE-derived markers provided a different type of information. Static SPE fractions, including albumin, alpha-1, and alpha-2, did not differ significantly between SD and CSU. IPR was originally proposed by Antonucci et al. as an SPE-derived marker of inflammatory status, based on the relationship between albumin and alpha fractions (8). It was slightly lower in SD, but the difference was not statistically significant, and it was not associated with the diagnostic group after age and sex adjustment. This argues against IPR as a useful marker for distinguishing SD from CSU. At the same time, IPR correlated strongly with CRP in both SD- and CSU-only patients, and albumin correlated inversely with CRP in both groups. Thus, IPR appears to reflect general inflammatory coupling with CRP rather than subtype-specific inflammation. The current study did not address qualitative SPE interpretation because monoclonal peaks, atypical bands, and immunofixation results were not systematically analyzed.

Anti-TPO positivity was numerically less frequent in SD than in CSU and was associated with higher odds of CSU in the adjusted analysis. This direction is compatible with the known autoimmune signal in CSU and with the findings of Can and Kocatürk, who reported higher anti-TPO positivity in CSU than in SD (13). However, thyroid autoimmunity was not a central focus of this study and should be interpreted as a secondary finding.

The main strength of this study is its focus on a practical, guideline-related question in a real-world SD cohort: whether low-cost inflammatory markers provide interpretable information in SD. Additional strengths include the clearly defined SD- and CSU-only groups, the use of CSU as a clinically related reference comparator, and inclusion of exploratory SPE-derived markers. The partly negative findings are clinically important because they help define the limits of routine laboratory testing and reduce the risk of overinterpreting nonspecific markers.

Several limitations should be acknowledged. The study was retrospective and based on routinely available laboratory data, with missing values for some markers. ESR was not available, so the full ESR-or-CRP option recommended in the international urticaria guideline could not be evaluated (4). Treatment status, treatment response, and provocation-threshold data were not collected, which limited the interpretation of laboratory markers in relation to disease activity and clinical course. The age- and sex-adjusted logistic regression models were exploratory, assessed individual markers separately, and were not intended to provide a multivariable prediction model or a formal evaluation of predictive performance. Finally, qualitative SPE findings relevant to monoclonal gammopathy or atypical inflammatory patterns were not systematically collected.

Overall, these findings show that routine inflammatory markers can provide some interpretable information in SD, but do not clearly explain why they should be measured routinely. CRP and the differential blood count may still be useful when clinically indicated to identify systemic inflammation or signal the need to consider alternative diagnoses, whereas SPE-derived markers should remain exploratory or be reserved for selected atypical presentations.

## Data Availability

The datasets analyzed during the current study are not publicly available because they contain clinical real-world data but may be available from the corresponding author upon reasonable request and subject to institutional and ethical approvals. Requests to access the datasets should be directed to Mojca Bizjak-Suran, mojca.bizjak@klinika-golnik.si.
