# Supplementary material for: Guideline-recommended inflammatory testing in symptomatic dermographism: a real-world study
Source: Front Immunol. 2026 Aug 6;17:1901562. doi: 10.3389/fimmu.2026.1901562 (PMC13490054; doi:10.3389/fimmu.2026.1901562)
Supplement: Supplementary file 1 [file Table1.docx]

**Supplementary Table S1. Full laboratory comparison between SD-only and CSU-only patients**

| **Parameter** | **Overall**  **n=686** | **SD-only**  **n=107** | **CSU-only**  **n=579** | ***P* value** |
| --- | --- | --- | --- | --- |
| **Demographics** |  |  |  |  |
| Age (y) | 45.0 (35.0–57.0) | 39.0 (32.0–49.0) | 45.0 (35.0–58.0) | **0.001** |
| Female gender | 453 (66.0%) | 77 (72.0%) | 376 (64.9%) | 0.183 |
| **Complete blood count** |  |  |  |  |
| Leukocytes (×10⁹/L) | 6.64 (5.57–8.12), n=445 | 6.66 (5.61–8.02), n=79 | 6.64 (5.56–8.21), n=366 | 0.701 |
| Leukocytosis (>10.00 ×10^9^/L) | 36 (8.1%), n=445 | 3 (3.8%), n=79 | 33 (9.0%), n=366 | 0.171 |
| Neutrophils (×10⁹/L) | 4.00 (3.26–5.24), n=445 | 3.93 (3.03–5.05), n=79 | 4.04 (3.30–5.24), n=366 | 0.197 |
| Neutrophilia (>7.40 ×10^9^/L) | 26 (5.8%), n=445 | 1 (1.3%), n=79 | 25 (6.8%), n=366 | 0.063 |
| Lymphocytes (×10⁹/L) | 1.79 (1.44–2.22), n=445 | 1.90 (1.52–2.24), n=79 | 1.76 (1.42–2.22), n=366 | 0.209 |
| Eosinophils (×10⁹/L) | 0.12 (0.07–0.20), n=445 | 0.14 (0.09–0.22), n=79 | 0.12 (0.07–0.20), n=366 | 0.188 |
| Basophils (×10⁹/L) | 0.03 (0.02–0.05), n=445 | 0.04 (0.03–0.05), n=79 | 0.03 (0.02–0.05), n=366 | **<0.001** |
| **SPE** |  |  |  |  |
| Albumin (g/L) | 43.60 (41.68–45.40), n=654 | 43.55 (41.63–45.40), n=100 | 43.60 (41.68–45.43), n=554 | 0.852 |
| Alpha-1 (g/L) | 2.90 (2.60–3.20), n=654 | 2.80 (2.60–3.10), n=100 | 2.90 (2.60–3.20), n=554 | 0.058 |
| Alpha-2 (g/L) | 6.90 (6.30–7.60), n=654 | 6.80 (6.10–7.50), n=100 | 7.00 (6.30–7.70), n=554 | 0.117 |
| Beta-1 (g/L) | 4.40 (4.00–4.80), n=654 | 4.45 (4.03–4.80), n=100 | 4.40 (4.00–4.80), n=554 | 0.437 |
| Beta-2 (g/L) | 3.40 (2.90–3.80), n=654 | 3.30 (2.90–3.70), n=100 | 3.40 (2.90–3.90), n=554 | 0.392 |
| Gamma (g/L) | 10.50 (9.10–12.00), n=654 | 10.70 (9.70–12.30), n=100 | 10.50 (9.00–12.00), n=554 | 0.163 |
| Albumin/globulin ratio | 1.55 (1.40–1.68), n=654 | 1.56 (1.42–1.66), n=100 | 1.54 (1.39–1.68), n=554 | 0.956 |
| **Other parameters** |  |  |  |  |
| CRP (mg/L) | 2.00 (0.80–5.10), n=603 | 1.40 (0.70–3.30), n=99 | 2.10 (0.90–5.50), n=504 | **0.003** |
| High CRP (≥5 mg/L) | 153 (25.4%), n=603 | 16 (16.2%), n=99 | 137 (27.2%), n=504 | **0.023** |
| Total serum protein (g/L) | 71.95 (69.40–74.93) | 72.20 (69.30–74.90) | 71.90 (69.40–75.00) | 0.852 |
| Serum IgA (g/L) | 2.05 (1.50–2.80), n=192 | 2.00 (1.55–2.60), n=60 | 2.10 (1.50–2.80), n=132 | 0.665 |
| Serum IgG (g/L) | 11.70 (10.20–13.05), n=189 | 11.80 (10.80–13.00), n=59 | 11.65 (9.88–13.10), n=130 | 0.451 |
| Serum IgM (g/L) | 1.10 (0.75–1.50), n=189 | 1.20 (0.80–1.60), n=59 | 1.00 (0.70–1.50), n=130 | 0.085 |
| Anti-TPO (IU/mL) | 15.00 (10.00–19.00), n=455 | 15.00 (9.00–15.00), n=78 | 15.00 (10.00–19.50), n=377 | 0.398 |
| Anti-TPO positivity (≥34 IU/mL) | 74 (16.3%), n=455 | 7 (9.0%), n=78 | 67 (17.8%), n=377 | 0.063 |
| **Derived inflammatory markers** |  |  |  |  |
| NLR | 2.33 (1.77–3.04), n=445 | 2.15 (1.55–2.79), n=79 | 2.35 (1.78–3.12), n=366 | **0.043** |
| High NLR (≥2.5) | 179 (40.2%), n=445 | 28 (35.4%), n=79 | 151 (41.3%), n=366 | 0.377 |
| PLR | 145.59 (117.85–182.78), n=445 | 143.65 (112.50–186.84), n=79 | 145.86 (119.10–181.97), n=366 | 0.604 |
| MLR | 0.29 (0.23–0.36), n=445 | 0.28 (0.22–0.37), n=79 | 0.29 (0.23–0.36), n=366 | 0.532 |
| SII | 601.4 (435.3–807.1), n=445 | 533.12 (410.06–818.75), n=79 | 614.72 (441.84–801.75), n=366 | 0.194 |
| SIRI | 1.19 (0.84–1.72), n=445 | 1.06 (0.74–1.64), n=79 | 1.23 (0.87–1.72), n=366 | 0.155 |
| CRP-to-albumin ratio | 0.05 (0.02–0.12), n=576 | 0.03 (0.02–0.08), n=93 | 0.05 (0.02–0.13), n=483 | **0.007** |
| IPR (%) | 22.38 (20.28–25.42), n=654 | 21.59 (19.37–25.27), n=100 | 22.47 (20.51–25.45), n=554 | 0.097 |

Data are presented as median (interquartile range) or n (%). Where data were not available for all patients, the number of patients with available data is shown as n. Definitions of derived inflammatory markers are provided in the Methods. *P* values were calculated using Fisher’s exact test for categorical variables and the Mann–Whitney U test for continuous variables.

*Abbreviations*: anti-TPO, anti-thyroid peroxidase antibodies; CBC, complete blood count; CRP, C-reactive protein; CSU, chronic spontaneous urticaria; IPR, inflammatory protein ratio; IQR, interquartile range; MLR, monocyte-to-lymphocyte ratio; NLR, neutrophil-to-lymphocyte ratio; PLR, platelet-to-lymphocyte ratio; SD, symptomatic dermographism; SII, systemic immune-inflammation index; SIRI, systemic inflammation response index; SPE, serum protein electrophoresis.
